# Novel retinoic acid metabolism blocking agents have potent inhibitory activities on human breast cancer cells and tumour growth

**DOI:** 10.1038/sj.bjc.6603705

**Published:** 2007-03-27

**Authors:** J B Patel, J Mehta, A Belosay, G Sabnis, A Khandelwal, A M H Brodie, D R Soprano, V C O Njar

**Affiliations:** 1 Department of Pharmacology and Experimental Therapeutics, University of Maryland School of Medicine, 655 West Baltimore Street, Baltimore, MD 21201-1559, USA; 2 The University of Maryland Marlene and Stewart Greenebaum Cancer Center, School of Medicine, Baltimore, MD 21201-1559, USA; 3 Department of Biochemistry, Temple University School of Medicine, 3420 North Broad Street, Philadelphia, PA 19140, USA

**Keywords:** breast cancer, retinoic acid, RAMBAs, differentiation, apoptosis, antitumour activity

## Abstract

Antitumour effects of retinoids are attributed to their influence on cell proliferation, differentiation, apoptosis and angiogenesis. In our effort to develop useful agents for breast cancer therapy, we evaluated the effects of four representative retinoic acid metabolism blocking agents (RAMBAs, VN/14-1, VN/50-1, VN/66-1 and VN/69-1) on growth inhibition of oestrogen receptor positive (ER +ve, MCF-7 and T-47D) and oestrogen receptor negative (ER −ve, MDA-MB-231) human breast cancer cells. Additionally, we investigated the biological effects/molecular mechanism(s) underlying their growth inhibitory properties as well as their antitumour efficacies against MCF-7 and MCF-7Ca tumour xenografts in nude mice. We also assessed the effect of combining VN/14-1 and all-*trans*-retinoic acid (ATRA) on MCF-7 tumuor xenografts. The ER +ve cell lines were more sensitive (IC_50_ values between 3.0 and 609 nM) to the RAMBAs than the ER −ve MDA-MB-231 cell line (IC_50_=5.6–24.0 *μ*M). Retinoic acid metabolism blocking agents induced cell differentiation as determined by increased expression of cytokeratin 8/18 and oestrogen receptor-*α* (ER-*α*). Similar to ATRA, they also induced apoptosis via activation of caspase 9. Cell cycle analysis indicated that RAMBAs arrested cells in the G1 and G2/M phases and caused significant downregulation (>80%) of cyclin D1 protein. *In vivo*, the growth of MCF-7 mammary tumours was dose-dependently and significantly inhibited (92.6%, *P*<0.0005) by VN/14-1. The combination of VN/14-1 and ATRA also inhibited MCF-7 breast tumour growth *in vivo* (up to 120%) as compared with single agents (*P*<0.025). VN/14-1 was also very effective in preventing the formation of MCF-7Ca tumours and it significantly inhibited the growth of established MCF-7Ca tumours, being as effective as the clinically used aromatase inhibitors, anastrozole and letrozole. Decrease in cyclin D1 and upregulation of cytokeratins, Bad and Bax with VN/14-1 may be responsible for the efficacy of this compound in inhibiting breast cancer cell growth *in vitro* and *in vivo*. Our results suggest that our RAMBAs, especially VN/14-1 may be useful novel therapy for breast cancer.

The potential of all-*trans*-retinoic acid (ATRA) and other retinoids in prevention and therapy of a number of proliferative diseases including breast cancer was noted almost 20 years ago (Moon *et al*, 1985). The ability of ATRA as well as other natural and synthetic retinoids to modulate a variety of important functions such as cell growth and differentiation, induction of apoptosis and prevention of angiogenesis is well documented (Dragnev *et al*, 2000; Altucci and Gronemeyer, 2001; Boyle, 2001; Fontana and Rishi, 2002). 4-Hydroxyphenyl retinamide (4-HPR) – a synthetic retinoid – is a possible therapeutic option for early breast cancer in premenopausal women (Torrisi and Decensi, 2000).

All-*trans*-retinoic acid is currently being used in the treatment of acne and psoriasis. It is effective in chemotherapy of acute promyelocytic leukaemia and also inhibiting the *in vivo* development of carcinogen-induced carcinoma of the breast, bladder, liver, lung, pancreas, prostate, ovaries and skin (Miller, 1998; Altucci and Gronemeyer, 2001; Njar *et al*, 2006a, 2006b). All-*trans*-retinoic acid and its isomers exert their action by binding to their nuclear receptors: retinoic acid receptors (RARs) and retinoid X receptors (RXRs). Both the receptors have three subtypes *α*, *β* and *γ* that regulate the expression of a variety of genes. These receptors also heterodimerise with other nuclear steroid receptors that lead to transcription of various genes.

Although numerous studies have shown the anticancer effect of ATRA against different neoplasms, the clinical use of ATRA has been thwarted by the emergence of resistance (Wouters *et al*, 1992; Van Huesden *et al*, 2002). Several molecular mechanisms may underlie ATRA resistance, including (1) downregulated expression or mutation of RAR*α* or -*β* receptors (Lin *et al*, 2000; Tanaka *et al*, 2004); (2) different levels of expressions of cellular retinoic acid binding proteins (CRABP I and II) (Arapshian *et al*, 2004) leading to rapid metabolism of ATRA (Budhu and Noy, 2002); (3) overexpression of HER2/Grb2/Akt pathway (Mendoza-Gamboa *et al*, 2004); and (4) metabolism of ATRA by cytochrome *P*450 (CYP)-dependent ATRA 4-hydoxylase enzymes namely CYP26 family, CYP2C8 and CYP3A4 (McSorley and Daly, 2000; Marill *et al*, 2002; Njar, 2002; Njar *et al*, 2006a, 2006b). All-*trans*-retinoic acid is responsible for inducing the expression of cytochrome *P*450 enzymes leading to its catabolism. The physiologically most prominent pathway for ATRA metabolism starts with the hydroxylation at the C-4 position of the cyclohexenyl ring leading to the formation of 4-hydroxy-ATRA and other metabolic by-products. This tightly controlled negative feedback mechanism limits the availability of ATRA and consequently its biological activity. Therefore, the strategy to increase intracellular levels of ATRA by inhibition of its metabolism is considered an innovative approach for cancer therapy. Inhibitors of ATRA metabolism are also called retinoic acid metabolism blocking agents (RAMBAs).

Whereas several categories of nonretinoidal RAMBAs have been reported (reviewed in Njar, 2002; Njar *et al*, 2006a), only a few retinoidal RAMBAs developed by our group are known. Our RAMBAs are also considered to be *atypical*, because in addition to being potent inhibitors of ATRA metabolism (able to enhance the antiproliferative action of ATRA), they also possess intrinsic potent cancer antiproliferative activities (Njar *et al*, 2000, 2006a; Njar, 2002; Patel *et al*, 2004; Huynh *et al*, 2006; Belosay *et al*, 2006). We have also found that our RAMBAs appear to have different potencies in different types of human cancer cell lines. For example, VN/14-1 is a weak inhibitor of several prostate cancer cells but the compound has been identified as our most potent RAMBA in several breast cancer cell lines (Patel *et al*, 2004; Belosay *et al*, 2006; Huynh *et al*, 2006). Here, we present data on growth inhibition, molecular mechanisms/biological effects and *in vivo* antitumour effects of these new RAMBAs against breast cancer cell lines and tumours. We report that our RAMBAs are capable of inducing differentiation, apoptosis, affecting the cell cycle proteins, as well as altering the expression of oestrogen receptor-*α* (ER-*α*) in breast cancer cells. In addition, we have identified VN/14-1 as a highly potent suppressor of growth of human MCF-7 and MCF-7Ca tumour xenograft (derived from MCF7Ca cells, that is human MCF-7 human breast cancer cells stably transfected with human aromatase gene) in female nude mouse model that is more effective than either ATRA or 4-HPR. VN/14-1 is also as effective as clinically used aromatase inhibitors (AIs) and caused prevention of MCF-7Ca tumour formation.

## MATERIALS AND METHODS

### Material for cell culture

Improved Dulbecco's modified Eagle's medium (IMEM), RPMI 1640 medium, OPTI-MEM I (low-serum medium), Dulbecco's phosphate-buffered saline (PBS), trypsin/EDTA solution and penicillin/streptomycin were purchased from GIBCO (Invitrogen Corporation, Grand Island, NY, USA). Regular fetal bovine serum (FBS) was from Hyclone (Logan, UT, USA). Tissue culture flasks (T-25, T-75 and T-150), six-well plates and 24-well plates were obtained from Corning Incorporated (Corning, NY, USA).

### Cell lines

Hormone-dependent/oestrogen receptor positive (ER +ve) MCF-7 cells were a generous gift from Dr Richard Santen (University of Virginia health system, Charlottesville, VA, USA), whereas MDA-MB-231 (oestrogen receptor negative (ER –ve)) cells were purchased from American type cell culture (ATCC). MCF-7 and MDA-MB-231 cells were cultured in IMEM with glutamine and phenol red, supplemented with 5% FBS and 1% penicillin–streptomycin. MCF-7Ca cells were cultured in Dulbeco's modified Eagle's medium supplemented with 5% FBS, 1% penicillin–streptomycin, 700 *μ*g ml^−1^ G_418_. Cells were grown as a monolayer in T75 or T150 tissue culture flasks in a humidified incubator (5% CO_2_, 95% air) at 37°C. Hormone-dependent/ER +ve T47D cells were purchased from ATCC, and cultured in RPMI 1640 medium with glutamine, supplemented with 10% FBS and 1% penicillin–streptomycin. Cells were grown as a monolayer in T75 or T150 tissue culture flasks in a humidified incubator (5% CO_2_, 95% air) at 37°C.

### Chemicals/test compounds

**Figure 1:**
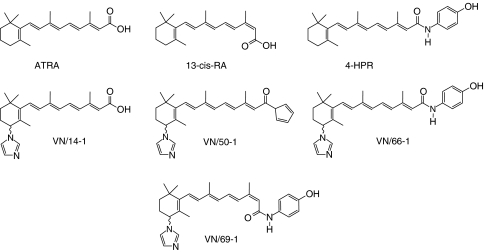
Structures of retinoic acids, RAMBAs and retinoid 4-HPR.

All-*trans-*retinoic acid was purchased from Sigma Chemical Company (St Louis, MO, USA) and LKT laboratories Inc. (St Paul, MN, USA), whereas [11,12-^3^H]ATRA was purchased from Perkin Elmer Life Sciences Inc. (Boston, MA, USA). Retinoic acid metabolism blocking agents (VN/14-1, VN/50-1, VN/66-1 and VN/69-1) (Figure 1) were designed and synthesised by us (Njar *et al*, 2000; Patel *et al*, 2004). 4-Hydroxyphenyl retinamide (fenretinide) is available commercially, but was also synthesised in our laboratory. 4-Hydroxyphenyl retinamide was prepared by a literature method (Moon *et al*, 1979), provided spectral and analytical data as described (Moon *et al*, 1979).

### Antibodies for Western immunoblotting

Antibodies against cytokeratin 8/18 (CK 8/18), E-cadherin and ER-*α* were purchased from Santa Cruz Biotechnology Inc. (Santa Cruz, CA, USA) Antibodies against poly ADP ribose polymerase (PARP), Cyclin- D1, Caspase-9 and Bad were obtained from Cell Signaling Technology (Danvers, MA, USA). Antibody for *β*-actin was obtained from Calbiochem (San Diego, CA, USA). Secondary anti-mouse IgG horseradish peroxidase (HRP) conjugate antibody was purchased from Calbiochem and Bio-Rad. Secondary anti-rabbit IgG HRP conjugate antibody was purchased from Bio-Rad as well as from Kirkegaard and Perry laboratories.

### Animals and material for *in vivo* studies

Ovariectomised female athymic nude mice (4–6-week old) used for tumour xenograft studies were obtained from NCI/FDRC. The mice were maintained in controlled environment with food and water *ad libitum*. Oestrogen pellets (1.7 mg per pellet, 90 day release) were purchased from Innovative Research of America (Sarasota, FL, USA). Matrigel was purchased from BD Biosciences (Bedford, MA, USA). All the animal studies were performed according to the guidelines and approval of the animal care committee of the University of Maryland School of Medicine, Baltimore, and were consistent with United Kingdom Coordinating Committee on Cancer Research guidelines for the welfare of animals in experimental neoplasia.

### Cell growth inhibition assays (MTT colorimetric assay)

These assays were performed as described previously (Patel *et al*, 2004).

### Retinoid receptor binding assays

To determine the IC_50_ values for each RAMBA, competition binding experiments were performed as described previously (Soprano *et al*, 2000; Zhang *et al*, 2003). The full-length cDNA clones for RAR*α*, RAR*β*, RAR*γ* and RXR*α* were cloned into the prokaryotic expression vector pET29 and recombinant S-Tag protein for each RAR subtype, and RXR were prepared as described previously (Tairis *et al*, 1995; Scafonas *et al*, 1997). Competition binding experiments were performed using a single concentration of 1.0 nM [^3^H]-all-*trans*-RA (1.82–1.92 TBq mmol^−1^ or 49.2–52.0 Ci mmol^−1^; Dupont (Boston, MA, USA) NEN) for RAR*α*, RAR*β* and RAR*γ* or 1.0 nM [^3^H]-9-*cis*-RA (1.74 TBq mmol^−1^ or 47.2 Ci mmol^−1^; Amersham, Piscataway, NJ, USA) for RXR*α* and various concentrations of each RAMBA ranging from 1 nM to 1 *μ*M. IC_50_ values are the concentration of each VN compound that reduced binding of either [^3^H]-all-*trans*-RA (RARs) or [^3^H]-9-*cis*-RA (RXR*α*) by 50%.

### Transactivation assays

The ability of each RAMBA to function as a tanscriptional agonist of RAR*α*, RAR*β* and RAR*γ* was determined as described previously (Tairis *et al*, 1995; Scafonas *et al*, 1997; Soprano *et al*, 2000; Zhang *et al*, 2003). Briefly, CV-1 cells were cotransfected with 4 *μ*g RAR subtype (RAR*α*, RAR*β* or RAR*γ*) expression vector construct in pSG5, 4 *μ*g RARE-CAT reporter DNA and 1 *μ*g pCMV-*β*-gal DNA. Twenty-four hours following transfection, the cells were treated with various concentrations of each RAMBA ranging from 10^−11^–10^−5^ M along with an ethanol carrier control. Additional cells were treated with 10^−6^ M all-*trans*-RA for normalisation. Twenty-four hours later, the cells were harvested and CAT and *β*-gal activities were assayed. The EC_50_ value for each RAMBA represents the concentration of the compound that results in 50% of the maximal activity obtained with 10^−^6 M all-*trans*-RA.

### Anti-AP1 assay

The ability of each RAMBA to inhibit AP1 activity was determined using anti-AP1 assays as we have described previously (Soprano *et al*, 2000). Briefly, CV-1 cells were cotransfected with 1.5 *μ*g RAR subtype (RAR*α*, RAR*β* and RAR*γ*) expression vector construct in pSG5, 1.5 *μ*g AP-1 CAT reporter DNA and 0.5 *μ*g pCMV-*β*-gal DNA. Four hours following transfection, the cells were treated with one of the following treatments: ethanol alone, 10^−^6 M ATRA, 10^−^6 M VN/14-1 or 10^−6^ M ATRA+10^−6^ M VN/14-1. Twenty-four hours later, the cells were harvested and CAT and *β*-gal activities were assayed. The percentage of AP-1 activity was calculated using the ethanol carrier control sample as 100%.

### CRABP binding assays

Wild-type full-length cDNAs for mouse CRABPI and CRABPII were subcloned into the prokaryotic expression vector pRSETB and transformed into *Escherichia coli* strain BL21(DE3)pLys (Chen *et al*, 1995). Recombinant fusion proteins were prepared essentially as we have described previously using Ni-NTA resin (Chen *et al*, 1995). The binding of each RAMBA to CRABPI and CRABPII was determined by competition binding assays as described previously (Chen *et al*, 1995). Briefly, 50 nM CRABPI or CRABPII protein was incubated with 5 or 25 nM [^3^H]-all-*trans*-RA (1.82–1.92 TBq mmol^−1^ or 49.2–52.0 Ci mmol^−1^; Dupont NEN) (near the *K*_d_ value for CRABPI or CRABPII, respectively) and various concentrations of each RAMBA ranging from 1 to 500 nM. IC_50_ values were calculated as described above in the RAR binding studies.

### Determination of apoptosis in breast cancer cells

#### TUNEL assay using *in situ* cell death detection kit, alkaline phosphatase

Breast cancer cells (5000 cells per chamber) were plated on an eight-chamber slide (Nunc lab-Tek chamber slide system (Fisher Scientific, Pittsburgh, PA, USA). Cells were allowed to adhere for about 24 h and then treated with different concentrations (1 and 5 *μ*M) of ATRA or RAMBAs. The medium and drug were renewed on day 3. After 6 days of treatment, the medium was aspirated; cells were washed with PBS and then fixed with 4% para formaldehyde for about 45 min on the slides. After fixing, the cells were washed with PBS and stored at 4°C until further staining. The cells were then permeabilised using permeabilisation solution containing 0.1% Triton X-100 in 0.1% sodium citrate for 2 min on ice and then treated with the TUNEL (terminal deoxynucleotidyl transferase (TdT)-mediated dUTP nick-end-labelling) reaction mixture containing the enzyme terminal transferase and the label solution for 60 min at 37°C in a humidified atmosphere in the dark according to the protocol of *in situ* cell death detection kit, AP (Roche Diagnostics, Basel, Switzerland, Germany). The cells were rinsed with PBS and then the stained-labelled apoptotic cells were mounted using DAPI (diamidino-2-phenylindole 2HCl)-glycerol and visualised with a fluorescent microscope using two filters (1) for TUNEL stained cells, and (2) for DAPI stained cells, which represents all the cells (nuclei). Fluorescent microscope model was Nikon, Eclipse E400 and the software was SPOT advanced version 3.5.5 by Diagnostic Instruments Inc. (Sterling Heights, MI, USA).

### Preparation of cell lysates

Breast cancer cells (MCF-7 and T47D) were treated with different concentration (1, 5 or 10 *μ*M) of ATRA or RAMBAs or untreated in T-75 or T-150 flasks for 6 days. At the end of the treatment, cells were scrapped and collected by washing with ice-cold PBS in a centrifuge tube. Cells were centrifuged at 2500 r.p.m. for 10 min, the pellet was again washed with ice-cold PBS and centrifuged. The cell pellet was then suspended in chilled cell lysis buffer (0.1 M Tris–HCl, 0.5% Triton X-100, protease inhibitor cocktail) and sonicated on ice for 10–15 s. The homogenate was kept on ice for 30 min and then centrifuged at 13 000 r.p.m. for 30 min. The supernatant was used as cell lysate. The cell lysates were stored at −80°C. Protein concentration of the cell lysates was determined using Bio-Rad protein assay reagents.

### Gel electrophoresis and Western immunoblotting

Protein lysates from MCF-7 and T47D cells were subjected to sodium dodecyl sulphate–polyacrylamide gel electrophoresis (SDS–PAGE) and immunoblotted for different proteins. Equal amounts of protein (25–50 *μ*g) were subjected to SDS–PAGE at 60 V for 3 h using the mini PROTEAN3 electrophoresis module assembly (Bio-Rad, Hercules, CA, USA), and transferred to nitrocellulose membranes (Hybond ECL, Amersham). Immunodetections were carried out using antibodies against CK 8/18 (1° antibody – 1 : 8000 in 10% milk PBST), E-cadherin (1° antibody – 1 : 1000 in 5% milk in TBST) and ER-*α* (1° antibody – 1 : 200 in 5% milk PBST) purchased from Santa Cruz Biotechnology Inc. Antibodies against PARP (1° antibody – 1 : 1000 in 5% milk TBST), cyclin- D1 (1° antibody – 1 : 2000 in 10% milk TBST), caspase-9 (1° antibody – 1 : 1000 in 10% milk TBST) and BAD (1° antibody – 1 : 1000 in 5% BSA TBST) were obtained from Cell Signaling Technology. Antibody for *β*-actin was obtained from Calbiochem (CA, USA). Secondary anti-mouse IgG HRP conjugate antibody was purchased from Calbiochem and Bio-Rad. Secondary anti-rabbit IgG HRP conjugate antibody was bought from Bio-Rad as well as from Kirkegaard and Perry laboratories (Gaithersburg, MD, USA). Immunoreactive bands were visualised using the enhanced chemiluminescence detection reagents and analysis system (Amersham Bioscences) according to the manufacturer's instructions and quantified by densitometry using ImageQuant software version 5.

### Cell cycle analysis

MCF-7 and T47D cells were plated in T-25 flasks and allowed to adhere for about 18 h. The cells were then synchronised by growing them in low-serum OPTI-MEM I medium for 2 days. Then the cells were treated in regular medium for 6 days with different concentrations (1 and 5 *μ*M) of ATRA or RAMBAs with renewal of drug and media on day 3. After 6-day treatment, the cells were trypsinised, washed twice with PBS, centrifuged at 2500 r.p.m. to obtain a cell pellet. The cells (about 1–2 × 10^6^) were fixed with 3 ml of 70% ethanol (by adding drop-wise and taking care that the cells are not clumped together). Cells were kept at −20°C until further analysis (at least overnight). Next, cells (∼1–2 × 10^6^) were stained with 1 ml of propidium iodide (PI) (50 *μ*g ml^−1^)/RNase (100 *μ*g ml^−1^) solution and after about half an hour the fluorescence of stained nuclei (15 000 events) was analysed by flow cytometry (Becton Dickinson FACScan Instrument and quantification by Modfit LT version 3.1 software) with excitation wavelength of 488 nm. The percentage of G1, S, G2/M and sub-G1 (apoptotic cells) in the population of treated and untreated cells were determined.

### *In vivo* antitumour studies

All the animal studies were performed according to the guidelines and approval of the Animal Care Committee of the University of Maryland, School of Medicine. Ovariectomised female athymic nude 4–6-week-old mice were used. Oestrogen pellets (Wen *et al*, 2000; Patel *et al*, 2004) were implanted in the dorsal interscapular region of the mice using a trochar to facilitate tumour growth. Ovariectomised mice were then inoculated with MCF-7 cells (2 × 10^6^ cells in Matrigel per tumour growth site) subcutaneously (s.c.) on the right and left flank. Tumour volumes were measured weekly with calipers. When the tumour volumes reached 200–300 mm^3^, the mice were grouped into control and treatment groups (*n*=5) (Yue *et al*, 1994; Wen *et al*, 2000; Patel *et al*, 2004). Tumour volumes were similar in each group at the start of the treatment. Retinoic acid metabolism blocking agents were formulated in 0.3% HPC (hydroxy propyl cellulose). We used doses equivalent to 5, 10 and 20 mg kg^−1^ of ATRA, that is 16.5 (VN/14-1), 33.0 (ATRA, VN/14-1, VN/50-1 and 4-HPR) and 66.0 *μ*mol kg^−1^ (VN/14-1 and VN/66-1), respectively, in 200 *μ*l of vehicle s.c. injection, once per day. Compounds were injected continuously for three days with a drug holiday of one day after every 3 days of treatment. Twice per week, the mice were weighed and tumours were measured using a caliper. Tumour volume was calculated according to the formula 4/3*πr*_1_^2^*r*_2_ (*r*_1_<*r*_2_). The tumour treatment study was continued for 6 weeks. At the end of 6 weeks, mice were killed and blood plasma, as well as tumours, were collected, weighed and stored at −80°C until analysis.

The second experiment was similar to that described above, but consisted of only four groups. When the tumour volume has reached about 100 mm^3^, the mice were randomly divided in to four groups of five mice each. The control group received vehicle, whereas the other three groups received ATRA (33.0 *μ*mol kg^−1^ day^−1^), VN/14-1 (16.5 *μ*mol kg^−1^ day^−1^) or ATRA (33.0 *μ*mol kg^−1^ day^−1^)+VN/14-1 (16.5 *μ*mol kg^−1^ day^−1^). These treatments continued for 36 days and the tumours were measured and processed as described above.

The third *in vivo* antitumour experiment was conducted with MCF-7Ca tumours. MCF-7Ca were provided by Dr S Chen (City of Hope, Duarte, CA, USA). The tumour xenografts were grown in mice as described previously (Yue *et al*, 1994). Subconfluent cells were scraped into Delbecco's phosphate-buffered saline, collected by centrifugation and resuspended in Matrigel (10 mg ml^−1^) at 2.5 × 10^7^ cells ml^−1^. Each mouse received s.c. inoculations in two sites per flank with 100 *μ*l of cell suspension. Mice in the tumour formation prevention group (*n*=5) were than injected daily with androstenedione (100 *μ*g day^−1^) plus VN/14-1 (20 mg kg^−1^ day^−1^) in the vehicle for the duration of the experiment. The rest of the mice were injected daily with 100 *μ*g day^−1^ of androstenedione. Measurements and treatments began when the tumours reached approximately 300 mm^3^, about 6 weeks after cell inoculation. Mice were assigned to groups for treatment so that there was no statistically significant difference in tumour volumes among the groups at the beginning of the treatment. Tumours were measured and volumes calculated as described above. Mice were than injected s.c. daily with the indicated agents: 100 *μ*g per mouse per day (5 × weekly) plus 10 *μ*g per mouse per day (5 × weekly) of letrozole or 200 *μ*g per mouse per day (5 × weekly) of anastrozole and 20 mg kg^−1^ day^−1^ (5 × weekly) of VN/14-1. The doses of letrozole, anastrozole, androstendione and VN/14-1 used are as determined previously (Long *et al*, 2002, 2004; Patel *et al*, 2004). The mice were treated for the indicated times, after which they were killed by decapitation and blood collected. Tumours and uteri were excised, cleaned, weighed and stored at −80°C for additional analyses.

### Statistical analysis

The statistical differences among the groups were analysed using Student's *t*-test on Sigma Plot 2000 software or Mann–Whitney *U*-test using GraphPad Prism 4 for tumour statistics. Differences were considered to be statistically significant when *P*<0.05.

## RESULTS

### Effects of RAMBAs on MCF-7, T-47D and MDA-MB-231 cell growth

**Figure 2:**
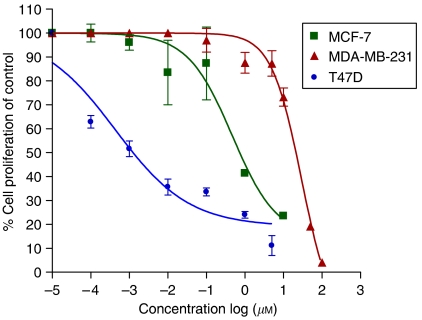
The effects of RAMBA VN/14-1 on proliferation of three human breast cancer cell line. For MCF-7 and T47D cells, 24-well plates were used and for MDA-MB-231 cells, 96-well plates were used. Cells (10 000 cells per well of a 24-well plate and 1000 cells per well of a 96-well plate) were plated and allowed to attach for 24 h. Cells were treated with VN/14-1 dissolved in 95% ethanol on day 1 and day 4 and analysed on day 7 by MTT assay using the spectrophotometer (Victor 1420 multi-label counter, Wallac (Perkin Elmer, Waltham, MA, USA)). For each concentration of the drug there were triplicate wells (24-well plate) and six wells (96-well plate) in every individual experiment. The data presented are mean±s.e.m. for 2–3 experiments. IC_50_ values were calculated by nonlinear regression analysis using GraphPad Prism software.

We have recently reported the synthesis, CYP26 inhibitions and effects of our novel RAMBAs on the growth of human breast and prostate cancer cell lines (Patel *et al*, 2004). These inhibitory effects of our lead RAMBAs (VN/14-1, VN/50-1, VN/66-1 and VN/69-1), ATRA and 4-HPR (Figure 1) on three breast cancer cells were further investigated in the present study with similar results. The data are summarised in Table 1, and reveal that these RAMBAs potently inhibited the growth of the two ER +ve human breast cancer cell lines (MCF-7 and T-47D) with IC_50_ values in the nanomolar range, with the T-47D cell being more sensitive. VN/14-1 is also a potent inhibitor of proliferation of MCF-7 cells stably transfected with human aromatase gene (MCF-7Ca) and also of the ER +ve letrozole resistant long-term letrozole cultured breast cancer cells (Belosay *et al*, 2006). The ER −ve MDA-MB-231 breast cancer cells were less sensitive to these agents (Table 1). The differential growth inhibitory effects of VN/14-1 are represented in Figure 2. These promising results with the ER +ve breast cancer cells prompted the present study, which examined the molecular mechanisms/biological effects underlying their growth inhibitory potencies. It should be stated that some of the procedure used in this study were identical to those utilised previously (Patel *et al*, 2004; Huynh *et al*, 2006). Except where otherwise stated, all cells were assessed following 6 days of ATRA/RAMBAs treatments. We point out that several researchers (Bollag *et al*, 1997; Toma *et al*, 1997) including us (Patel *et al*, 2004) have found that induction of several biochemical pathways in most cancer cell line become evident only after 6 days of ATRA/retinoid treatment.

### Effects of ATRA and RAMBAs on binding to CRABPs and retinoid receptors (RAR and RXR) and on transcriptional activation of RAR receptors

The ability of each of the four RAMBAs to bind CRABPs I and II and nuclear retinoid receptors was examined by competition bindings assays (Table 2). Only VN/14-1 displayed significant specific binding to each of the RAR subtypes with IC_50_ values ranging from 16 nM for both RAR*α* and RAR*γ* to 200 nM for RAR*β*. None of the four RAMBAs displayed binding to CRABPI, CRABPII and RXR*α*.

We then examined the functional activity of each of the four RAMBAs in transactivation assays with RAR*α*, RAR*β* and RAR*γ* (Table 2). Again only VN/14-1 induced RAR-dependent transcriptional activity with EC_50_ values of 300 nM for RAR*α*, 1500 nM for RAR*β* and 1000 nM for RAR*γ*. Since VN/14-1 displayed agonistic activity with each RAR subtypes, we examined the ability of VN/14-1 to inhibit AP1 activity. VN/14-1 at a concentration of 10^−6^ M displayed weak anti-AP1 activity reducing AP1-dependent transcription activity when each of the three RAR subtypes were cotransfected by 20–40%.

### Effect of ATRA, 4-HPR and RAMBAs on the expression levels of CK 8/18 and ER-*α* in MCF-7 and T47D cells

**Figure 3:**
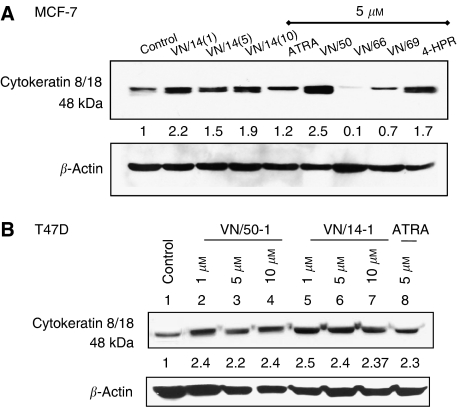
Western immunoblotting analysis of whole-cell lysates of treated MCF-7 and T47D cells for the expression of CK 8/18: (**A**) MCF-7 cells were treated with ATRA or RAMBAs for 6 days or 4HPR for 4 days and then cell lysates were electrophoresed using 10% SDS–PAGE and subjected to Western blotting. Lane 1: control; lanes 2–4: VN/14-1 (1, 5 and 10 *μ*M); lane 5: ATRA (5 *μ*M); lane 6: VN/50-1 (5 *μ*M); lane 7: VN/66-1 (5 *μ*M); lane 8: VN/69-1(5 *μ*M); and lane 9: 4-HPR (5 *μ*M). (**B**) T47D cells were treated with ATRA or RAMBAs (VN/50-1 and VN/14-1) for 6 days and then cell lysates were electrophoresed using 10% SDS–PAGE and subjected to Western blotting. Lane 1: control; lane 2: VN/50-1 (1 *μ*M); lane 3: VN/50-1 (5 *μ*M); lane 4: VN/50-1 (10 *μ*M); lanes 5–7: VN/14-1 (1, 5 and 10 *μ*M); and lane 8: ATRA (5 *μ*M). Numbers below the blot show fold increase in expression of the protein as analysed by ImageQuant densitometry analysis. Membranes were stripped and probed for *β*-actin to verify equal protein loading. Cytokeratin 8/18 is a 48 kDa protein. Primary antibody CK 8/18 (Santa Cruz Biotechnology) 1 : 8000 in 10% milk in PBST for 1 h, and secondary antibody (anti-mouse) 1 : 2000 in 10% milk in PBST for 1 h at room temperature. The experiments were repeated thrice with similar results.

The effects of these agents on the expression levels of markers associated with differentiation (CK 8/18 and ER-*α*) were investigated following established procedures (Jing *et al*, 1996; Korsching *et al*, 2002; Kim and Freeman, 2003; Woelfle *et al*, 2004). Breast cancer cells were treated with the indicated concentrations of ATRA or RAMBAs (VN/14-1, VN/50-1, VN/66-1 and VN/69-1) for 6 days or 4HPR for 4 days with renewal of media and compounds on day 3. At the end of the treatment, cell lysates were prepared and the expression of cytoskeletal proteins CK 8/18 were evaluated by Western immunoblotting. As shown in Figures 3A and B, expressions of CK 8/18 were increased in both MCF-7 and T47D cells after treatments with ATRA and RAMBAs. Treatments with 1 and 5 *μ*M concentrations of the compounds (ATRA, VN/14-1 and VN/50-1) increased ∼2–3–fold the expression of these proteins as compared with the control. However, no further up-modulation of theses markers was observed at higher concentrations of the RAMBAs. Unexpectedly, VN/66-1 and VN/69-1 caused downregulation in the expression of CK 8/18.

**Figure 4:**
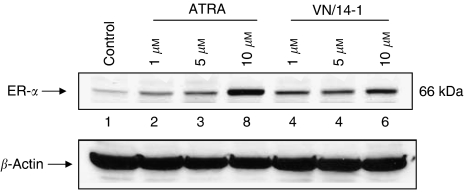
Western immunoblotting analysis of whole-cell lysates of treated MCF-7 cells for the expression of ER-*α*. MCF-7 cells were treated with ATRA and VN/14-1 for 6 days and then cell lysates were electrophoresed using 10% SDS–PAGE and subjected to Western blotting. Lane 1: control; lanes 2–4: ATRA (1, 5 and 10 *μ*M); and lanes 5–7: VN/14-1 (1, 5 and 10 *μ*M). Numbers below the blot show fold increase in expression of the protein as analysed by ImageQuant densitometry analysis. Membrane was stripped and probed for *β*-actin to verify equal protein loading. Primary antibody (Santa Cruz Biotechnology), 1 : 200 in 5% milk PBST for 2 h at RT, secondary antibody (anti-rabbit) 1 : 3000 in 5% milk PBST for 1 h at RT. The experiment was repeated twice with similar results.

An analogous set of experiments was performed to assess the effects of ATRA (1–10 *μ*M) and VN/14-1 (1–10 *μ*M) on the level of ER-*α* expression in MCF-7 cells. As demonstrated in Figure 4, there was a significant dose-dependent increase in the expression of ER-*α* elicited by both ATRA (from two- to eight-fold) and VN/14-1 (from four- to six-fold). Increase in ER is consistent with more differentiation. Together, these data suggest that the growth inhibitory effects of some of our RAMBAs may in part be due to their ability to induce differentiation in these two breast cancer cell lines.

### Effects of RAMBAs on cell cycle phase distribution and expression of cyclin D1

To further investigate the causes of the antiproliferative effects of RAMBAs, with ATRA and 4-HPR as positive controls, cell cycle analysis was performed on both MCF-7 and T47D cells treated with agents for 6 days. As described in Materials and Methods, cells were stained with PI and cell cycle analysis was performed by flow cytometry (Toma *et al*, 1997; Thiantanawat *et al*, 2003). Histograms were obtained from the analysis of both cell lines (data not shown) and the percentages of T47D cells in each cell cycle phase are presented in Table 3. The cell cycle profiles of the treated cells were more prominent in the T47D cells, as each treatment caused a significant increase in the percentage of cells in the G1 phase and decrease in the percentage of cells in the S phase, compared with the control. Among all treatments, VN/66-1 and VN/69-1 caused the greatest suppression (0%) of cells in the S phase, compared with 20.4% in the untreated control. These treatments also caused increases in the percentages of cells in the G2/M phase, but were more prominent in T47D than in the MCF-7 cells (data not shown). Furthermore, we also observed significant accumulation of cells in the sub-G1 phase of the treated cells, which may represent cells undergoing apoptosis (Table 3).

**Figure 5:**
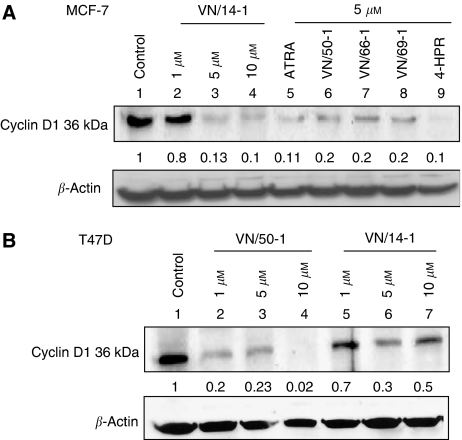
Western blotting of whole-cell lysates of treated MCF-7 and T47D cells for the expression of Cyclin D1. (**A**) MCF-7 cells were treated with ATRA or RAMBAs for 6 days or 4HPR for 4 days and then cell lysates were electrophoresed using 15% SDS–PAGE and subjected to Western blotting. Lane 1: control; lanes 2–4: VN/14-1 (1, 5 and 10 *μ*M); lane 5: ATRA (5 *μ*M); lane 6: VN/50-1 (5 *μ*M); lane 7: VN/66-1 (5 *μ*M); lane 8: VN/69-1(5 *μ*M); and lane 9: 4-HPR (5 *μ*M). (**B**) T47D cells were treated with VN/50-1 and VN/14-1 for 6 days and then cell lysates were electrophoresed using 15% SDS–PAGE and subjected to Western blotting. Lane 1: control; lanes 2–4: VN/50-1 (1, 5 and 10 *μ*M); and lanes 5–7: VN/14-1 (1, 5 and 10 *μ*M). Numbers below the blot show fold decrease in expression of the protein as analysed by ImageQuant densitometry analysis. Membranes were stripped and probed for *β*-actin to verify equal protein loading. Primary antibody (Cell Signaling Technology) 1 : 2000 in 10% milk TBST overnight at 4°C and secondary antibody (anti-rabbit) 1 : 2000 in 10% milk TBST for 1 h at RT. The experiments were repeated twice with similar results.

Given the well-established involvement of cyclins in the regulation of cell cycle progression and the previous findings that cyclin D1 is overexpressed in many cancers and cancer cell lines (Teixeira and Pratt, 1997; Zhou *et al*, 1997; Niu *et al*, 2001), we investigated the effects of our RAMBAs on the level of cyclin D1 expression. As shown in Figure 5A, ATRA, RAMBAs and 4-HPR, each significantly decreased cyclin D1 protein expression by >80% in MCF-7 cells as compared with untreated control. This decrease in the expression of cyclin D1 was also shown to be dose-dependent following treatment with VN/14-1. Similarly, the expression of cyclin D1 was significantly downregulated in T47D cells by treatments with VN/14-1 or VN/50-1 (Figure 5B).

### Apoptosis induced by RAMBAs

**Figure 6:**
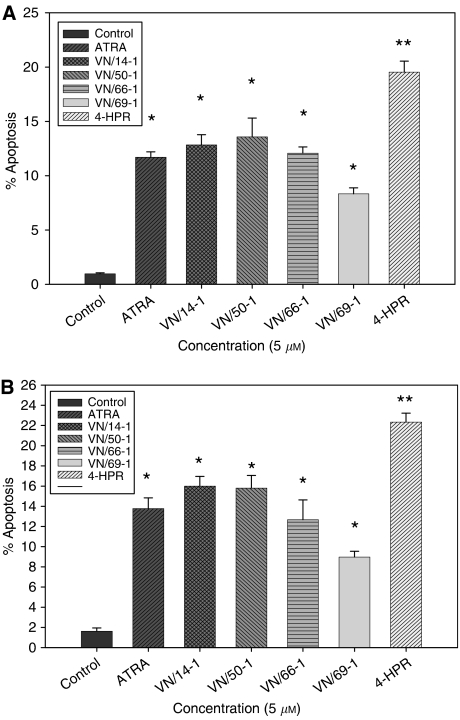
Graphs showing apoptosis induced in (**A**) MCF-7 and (**B**) T47D cells as determined by TUNEL. (**A**) MCF-7 cells and (**B**) T47D cells (4 × 10^4^)/ well were plated in eight-well slide and treated with 5 *μ*M of ATRA, RAMBAs and 4-HPR for 6 days (see Materials and Methods for details). TUNEL-stained apoptotic cells were counted against the DAPI stained cells to obtain percentage apoptosis. The experiments were repeated thrice with similar results. Error bars show s.e.m of three different fields of treated cells, statistically significant ^*^P<0.01 and ^**^P<0.001 *vs* control.

Because ATRA and most retinoids control cell proliferation via apoptosis among several other mechanisms in a variety of cancers (Bollag *et al*, 1997; Toma *et al*, 1997; Kotake-Nara *et al*, 2002; Afonja *et al*, 2004; Simeone and Tari, 2004) and also because of the cell cycle analysis data that suggests accumulation of cell in the sub-G phase, the apoptotic potential of our RAMBAs was evaluated. Retinoic acid metabolism blocking agents-induced apoptosis in MCF-7 and T47D cells was examined in three independent experiments with ATRA and 4-HPR as positive controls. First, TUNEL assays, involving a fluorescent DNA-binding dye, DAPI (Gavrieli *et al*, 1992; Thiantanawat *et al*, 2003) were used to study the morphology of dying cells following treatment with 5 *μ*M RAMBAs, ATRA or 4-HPR for 6 days. Cells undergoing apoptosis displayed the typical morphologic feature of apoptotic cells with condensed and fragmented nuclei, and in some cases, membrane blebbing (data not shown). Quantifications of apoptosis caused by the various agents are presented in Figure 6A and B. Following treatment with ATRA and RAMBAs, induction of apoptosis was 8–13% in MCF-7 cells (*P*<0.01) and 9–17% in T47D cells (*P*<0.01) as compared with control which had about 1.5% apoptosis. However, 4HPR caused the most induction of apoptosis in both the cell lines (20–25%; *P*<0.001 *vs* control). These results strongly suggest that our RAMBAs possess intrinsic apoptotic activity.

### Proteins involved in RAMBAs-induced apoptosis

To further characterise the apoptosis observed in the RAMBAs-treated breast cancer cells, we used Western blot analysis to assess the activation and cleavage of apoptosis-related proteins, including Bad, caspase 9 and PARP. As VN/14-1 was the most effective of the RAMBAs, we tested VN/14-1 in comparison to ATRA.

**Figure 7:**
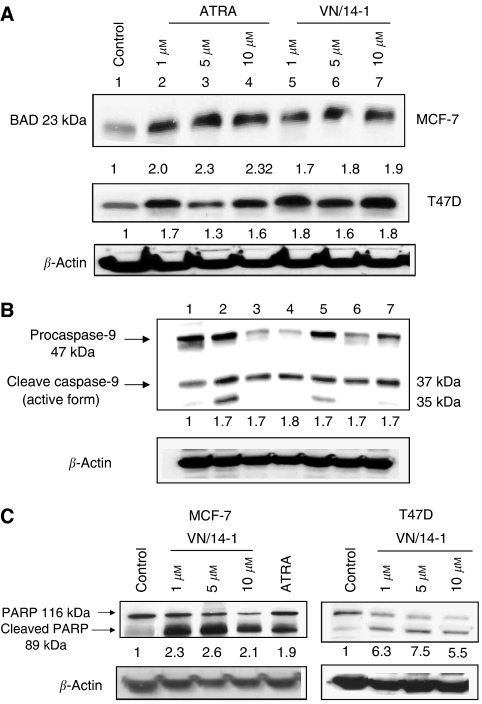
(**A**) Western immunoblotting of whole-cell lysates of treated MCF-7 and T47D cells for the expression of Bad. MCF-7 and T47D cells were treated with ATRA and VN/14-1 for 6 days and then cell lysates were electrophoresed using 15% SDS–PAGE and subjected to Western blotting. Lane 1: control; lanes 2–4: ATRA (1, 5 and 10 *μ*M); and lanes 5–7: VN/14-1 (1 5 and 10 *μ*M). Numbers below the blot show fold increase in expression of the protein as analysed by ImageQuant densitometry analysis. Membranes were stripped and probed for *β*-actin to verify equal protein loading. Primary antibody (Cell Signaling Technology) 1 : 1000 in 5% BSA TBST overnight at 4°C and secondary antibody (anti-rabbit) 1 : 2000 for 1 h at RT. This experiment was repeated twice with similar results. (**B**) Western blot showing activation of caspase-9 in T47D cells after treatment with ATRA or VN/14-1. T47D cells were treated with ATRA or VN/14-1 for 6 days and whole-cell lysates were electrophoresed using 10% SDS–PAGE and subjected to Western immunoblotting for caspase-9. Treatment with ATRA and VN/14-1 cleaved procaspase-9 to its active form. Lane 1: control; lanes 2–4: ATRA (1, 5 and 10 *μ*M); and lanes 5–7: VN/14-1 (1, 5 and 10 *μ*M). Numbers below the blot show fold increase in the active form of caspase-9 as compared with control. Membrane was stripped and probed for *β*-actin to verify equal amount of protein loading. The primary and secondary antibodies and their dilutions are as follows: primary antibody (Cell Signaling Technology), 1 : 1000 in 10% milk TBST overnight at 4°C and secondary antibody (anti-rabbit) 1 : 2000 in 10% milk TBST for 1 h at RT. Densitometry analysis was performed by ImageQuant software. This experiment was repeated twice with similar results. (**C**) Western immunoblotting of whole-cell lysates of treated MCF-7 and T47D cells for the expression of full-length and cleaved PARP. MCF-7 cells were treated with ATRA and RAMBAs for 6 days and 4-HPR for 4 days and then cell lysates were electrophoresed using 10% SDS–PAGE and subjected to Western blotting. Lane 1: control; lanes 2–4: VN/14-1 (1, 5 and 10 *μ*M); lane 5: ATRA (5 *μ*M); lane 6: VN/50-1 (5 *μ*M); lane 7: VN/66-1 (5 *μ*M); lane 8: VN/69-1(5 *μ*M); and lane 9: 4-HPR (5 *μ*M). T47D cells were treated with RAMBAs (VN/50-1 and VN/14-1) for 6 days and then cell lysates were electrophoresed using 10% SDS–PAGE and subjected to Western blotting. Lane 1: control; lanes 2–4: VN/50-1 (1, 5 and 10 *μ*M); lanes 5–7: VN/14-1 (1, 5 and 10 *μ*M). Numbers below the blot show fold increase in expression of the protein as analysed by ImageQuant densitometry analysis. Membranes were stripped and probed for *β*-actin to verify equal protein loading. Primary antibody (Cell Signaling Technology) 1 : 1000 in 5% milk TBST overnight at 4°C and secondary antibody (anti-rabbit) 1 : 2000 for 1 h at RT. The experiments were repeated twice with similar results.

We first investigated the effects of VN/14-1 on the expression of the pro-apoptotic Bad protein. Both VN/14-1 and ATRA at concentrations of 1, 5 and 10 *μ*M each induced upregulation of Bad protein by about two-fold, but there was no significant dose-dependent effect (Figure 7A). The possible involvement of Bad implies that induction of apoptosis by these treatments may be via the mitochondrial pathway. We therefore examined whether caspase-9 was activated. This molecule that plays a major role as an initiator caspase in this pathway was activated (Zou *et al*, 1999). As shown in Figure 7B, increased concentration of ATRA and VN/14-1 treatment caused an increase in the active form (37 kDa) and a decrease in the procaspase form (47 kDa). Control cells had a higher expression of the procaspase-9 (47 kDa) than the treated cells, that is 1 (control) *vs* 0.4- and 0.3-fold for 5 and 10 *μ*M ATRA, respectively, and 0.4- and 0.7-fold for 5 and 10 *μ*M of VN/14-1, respectively. The active form of caspase-9 was increased by 1.7- and 1.8-fold with 5 and 10 *μ*M of ATRA, respectively, and by 1.7- and 1.7-fold with 5 and 10 *μ*M of VN/14-1, respectively, compared with untreated control.

Another approach to detect apoptosis was by determining the proteolysis of PARP, the DNA repair enzyme that can be cleaved by effector caspases. As shown in Figure 7C, 5 *μ*M of ATRA, or VN/14-1 caused almost two-fold increases in the level of cleaved PARP (89 kDa) as compared with control in MCF-7 cells. Treatment of T47D cells (Figure 7C) with 1, and 5 *μ*M of VN/14-1 caused 6.3- and 7.5-fold increases, respectively, in the levels of cleaved PARP as compared with the control.

### *In vivo* antitumour study of ATRA, 4-HPR and RAMBAs

**Figure 8:**
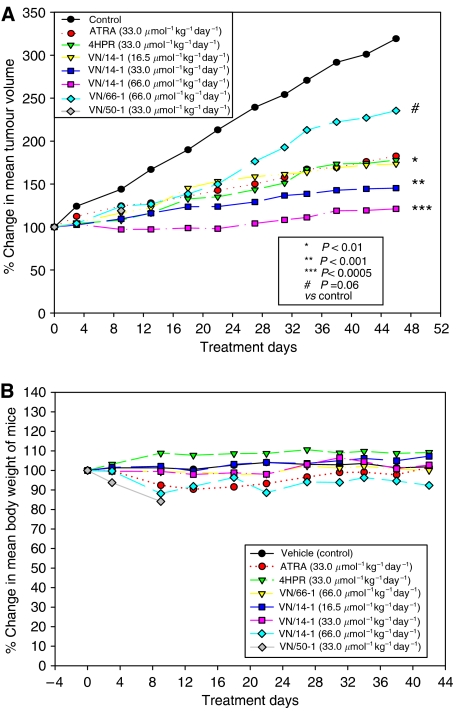
(**A**) Effect of ATRA, 4HPR or RAMBAs on the growth of MCF-7 tumour xenograft in ovariectomised female athymic nude mice. Ovariectomised female athymic nude 4–6-week-old mice were used. Oestrogen pellets (1.7 mg per pellet, 90 day release obtained from Innovative Research of America) were implanted in the mice using a trochar to facilitate tumour growth. Mice were then inoculated with MCF-7 cells (2 × 10^6^ cells in Matrigel per tumour growth site) s.c. on the right and left flank. Tumours were allowed to grow for about 4–5 weeks till they were of measurable size (200–300 mm^3^). The mice were then grouped as control and treatment groups. Twice every week the mice were weighed and tumours were measured using a caliper. Tumour volume was calculated according to the formula 4/3*πr*_1_^2^*r*_2_ (*r*_1_<*r*_2_). The tumour treatment study was continued for 6 weeks. (**B**) Effect of vehicle, ATRA 4-HPR and RAMBAs on body weight of ovariectomised female nude mice during the 6-week antitumour study. Mice were weighed twice every week during the 6-week antitumour study.

To assess the ability of RAMBAs to inhibit tumour growth, we examined their effects in human MCF-7 breast cancer xenograft model with ATRA and 4-HPR as positive controls. Female ovariectomised nude mice bearing established MCF-7 tumour xenografts (200–300 mm^3^) were grouped so that the mean tumour volume was similar for each group. They were then treated once daily, 6 days per week for a total of 6 weeks, with vehicle (control), ATRA (33.0 *μ*mol kg^−1^ day^−1^), 4-HPR (33.0 *μ*mol kg^−1^ day^−1^), VN/50-1 (33.0 *μ*mol kg^−1^ day^−1^), VN/66-1 (66.0 *μ*mol kg^−1^ day^−1^) and three doses of VN/14-1 (16.5, 33.0 and 66.0 *μ*mol kg^−1^ day^−1^). Tumour sizes were measured twice a week after the start of treatment. Because treatment with VN/50-1 was very toxic to the mice, the experiment with this cohort was terminated. As shown in Figure 8A, treatment with VN/14-1 produced a dose-dependent inhibition of tumour growth. Indeed, all three doses of VN/14-1, that is 16.5, 33.0 and 66.0 *μ*mol kg^−1^ day^−1^, caused significant reductions of 66.5% (*P*<0.01), 79.4% (*P*<0.001) and 92.6% (*P*<0.0005), respectively, in the mean tumour volume compared with the vehicle control. The effect of VN/14-1 (16.5 *μ*mol kg^−1^ day^−1^ equivalent to 5 mg kg^−1^ day^−1^ ATRA) was comparable with the antitumour effect of ATRA or 4-HPR (33.0 *μ*mol kg^−1^ day^−1^). VN/14-1 (33.0 and 66.0 *μ*mol kg^−1^ day^−1^ equivalent to 10 and 20 mg kg^−1^ day^−1^ of ATRA, respectively) caused a significant reduction of 45% (*P*<0.05) and 75.61% (*P*<0.005), respectively, in the mean tumour volume compared with the ATRA group (10 mg kg^−1^ day^−1^). The antitumour effect of VN/66-1 (66.0 *μ*mol kg^−1^ day^−1^) was not better than ATRA. Importantly, except for the VN/50-1-treated group, no significant changes in animal body weight were observed even at the highest concentration of VN/14-1 (Figure 8B), suggesting that the growth-inhibitory effects were tumour specific and that the treatments did not produce general cytotoxicity in the mice.

**Figure 9:**
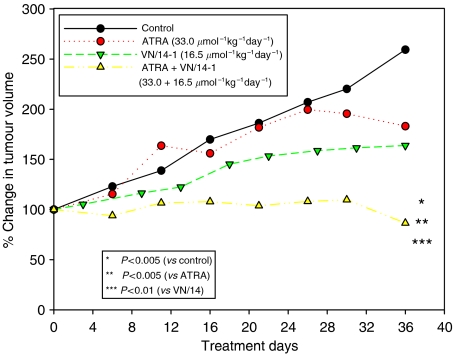
Effects of ATRA or VN/14-1 alone or in combination on the growth of MCF-7 tumour xenograft in ovariectomised female athymic nude mice. Procedure was similar to that described in Figure 8A.

It was of interest to determine the effects of combination of ATRA and VN/14-1 on the growth of MCF-7 tumour xenografts. Single agents ATRA (33.0 *μ*mol kg^−1^ day^−1^) or VN/14-1 (16.5 *μ*mol kg^−1^ day^−1^) resulted in a modest tumour growth inhibition compared with controls (Figure 9). However, the combination of ATRA and VN/14-1 resulted in a significant super additive inhibitory effect (up to 120% growth inhibition, as compared with controls; *P*<0.005 *vs* control). This result supports the hypothesis that RAMBA VN/14-1 is able to enhance the biological activity of ATRA through the inhibition of ATRA *in vivo*. We have demonstrated previously this effect in *in vitro* breast and prostate cancer models (Patel *et al*, 2004; Huynh *et al*, 2006).

### Effects of VN/14-1 compared with clinical AIs on growth of MCF-7Ca xenografts

**Figure 10:**
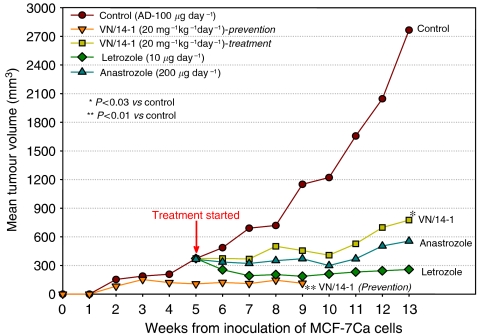
Effects of VN/14-1 on the formation of MCF-7Ca tumours and effects of VN/14-1, anastrozole and letrozole on the growth of MCF-7Ca xenografts in ovariectomised female athymic nude mice. Mice were inoculated with MCF-7Ca cells as described in Materials and Methods. Beginning on the following day, androstendione (100 *μ*g per mouse per day) was supplemented by s.c. injection for the duration of the experiment. For the tumour formation prevention group (*n*=5), VN/14-1 (20 mg kg^−1^ day^−1^) treatment began from the day after inoculation. For the other groups (VN/14-1, anastrazole and letrozole), treatment began after the tumour had reached approximately 300 mm^3^ following procedures described in Materials and Methods.

Based on our recent findings that VN/14-1 is also a potent AI (Belosay *et al*, 2006) and also because of the prominence of AIs in breast cancer therapy (Brodie *et al*, 2003), we wished to test the effects of VN/14-1 head to head with clinically used AIs, anastrozole or letrozole. As shown in Figure 10, VN/14-1 was as effective as either anastrozole or letrozole at inhibiting the growth of established MCF-7Ca tumours. In addition, VN14-1 was efficacious in preventing the formation of MCF-7Ca tumours (Figure 10). We also observed that VN/14-1 treatment caused significant reduction in uterine wet weight (data not shown), which indicates that VN/14-1 can effectively block the uterotropic activity of oestrogens produced by peripheral aromatisation of androstenedione (Goss *et al*, 2000; Jelovac *et al*, 2005).

## DISCUSSION

We have reported previously that our novel RAMBAs are potent inhibitors of ATRA metabolism and also potent inhibitors of proliferation of some human breast cancer cells (Patel *et al*, 2004). These results led us to propose that our RAMBAs also possess intrinsic retinoidal antitumour properties. Here, we have examined their molecular mechanisms of action, and shown that administration of some RAMBAs to nude mice bearing human oestrogen-dependent MCF-7 or MCF-7Ca breast tumours can effectively suppress tumour growth at doses that cause no apparent toxicity.

Like ATRA, each of the RAMBAs tested significantly caused up-modulation (two- to three-fold) of CK 8/18 in both MCF-7 and T47D breast cancer cell line, which suggest that our RAMBAs are capable of inducing differentiation in these breast cancer cell lines. Further support for the putative role of our RAMBAs as differentiating agents came from the findings that VN/14-1 caused a dose-dependent up-modulation (from four- to six-fold) of ER-*α* protein, another differentiation marker. The ability of these RAMBAs to induce differentiation in breast cancer cells may be of significance in light of recent findings which suggest that downregulated expressions of CK 18 (Korsching *et al*, 2002; Woelfle *et al*, 2004; Schaller and Buhler, 2005) and/or E-cadherin/ER-*α* (Nass *et al*, 2000; Kowalsski *et al*, 2003) promote progression of human breast cancer. It should be noted that differentiated tumour cells exhibit low proliferative and metastatic potential. An elevation in the expression of cytokeratins indicates a favourable prognosis and is a useful predictor for overall survival in breast cancer patients (Korsching *et al*, 2002). We have also shown that VN/14-1 binds and activates the RARs (RAR*α*, -*β* and -*γ*), but the other RAMBAs do not activate these receptors. Although it has been suggested that only ligands that activate RAR*α* can induce cell differentiation, our current results suggest that some of the RAMBAs are able to induce differentiation via RAR-independent pathway(s).

VN/14-1 binds (and transactivates) with higher affinity to RAR*α* and RAR*γ* compared with RAR*β*. Concentrations of VN/14-1 that can activate these two receptors will probably increase the level of CYP26 (along with other RA-inducible transcripts) in the cell. The question is how much of the antiproliferative effect of VN/14-1 is mediated by inhibition of CYP26 activity (increase in ATRA- and RAR-mediated transcription) *vs* some other mechanism leading to apoptosis that does not involve RARs.

Several studies have shown that retinoids inhibit proliferation of various cancers by inducing arrest of cells in a phase of the cell cycle. We have demonstrated here that our RAMBAs induce growth inhibition of both MCF-7 and T47D cells by G1 phase arrest with concomitant decrease of cells in S phase and also significant accumulation of cells in the sub-G1 phase. In addition, we also found increased percentage of cells in the G2/M, but this was consistently higher in the treated T47D cells than in the MCF-7 cells. We also demonstrated that expression of cyclin D1, the positive regulator of cell cycle progression from G1 to S phase, was reduced in MCF-7 and T47D cells after 6-day treatment with RAMBAs (1, 5 and 10 *μ*M). This result is in agreement with the cell cycle analysis, which revealed G1 phase cell arrest.

The ability of ATRA/retinoids to inhibit the growth of cells has previously been shown to result from their ability to induce apoptosis in various cancer cells. In the present investigation, a significant increase in cells in the sub-G1 phase, accounting for 10–20% of the total cell population was detected after exposure to RAMBAs. Cells in this phase are thought to represent cells that have undergone apoptosis. TUNEL analysis confirmed that the RAMBAs did induce a portion of cells (MCF-7 and T47D) to undergo apoptosis in a dose-dependent fashion.

Apoptosis induced by ATRA and RAMBAs was further characterised by assessing the activation and cleavage of apoptosis-related proteins as determined by Western blot analysis. Treatment of breast cancer cells (MCF-7 and T47D) with RAMBAs resulted in proteolysis of full-length PARP – the DNA repair enzyme, which was comparable to ATRA. In T47D cells, VN/14-1 was able to cleave pro-caspase-9 to active caspase-9, an effect similar to ATRA. However, the involvements of other caspases were not investigated. VN/14-1 and VN/50-1 also increased the expression of the pro-apoptotic Bcl-2 family member Bad. Thus, induction of apoptosis in breast cancer cells by the RAMBAs was confirmed by several methods. When compared with 4-HPR (a retinoid known for inducing apoptosis in breast cancer cells) (Pellegrini *et al*, 1995; Wu *et al*, 2001) apoptosis induced by RAMBAs was less than 4-HPR, but comparable to ATRA. Although VN/66-1 exhibited less antiproliferative activity, it induced apoptosis that was comparable to that induced by ATRA. VN/69-1 was the least effective of the RAMBAs in inducing apoptosis in both MCF-7 and T47D cells.

The marked effects that our RAMBAs had on cell proliferation led to an *in vivo* study. We studied the effects of VN/14-1, VN/50-1, VN/66-1 and ATRA. VN/50-1 was found to be toxic to the animals and its antitumour efficacy could not be assessed. Our results show that on a molar basis, ATRA and 4-HPR were each more effective in suppressing the growth of established MCF-7 tumours than VN/66-1, but both agents were each less effective than VN/14-1. Indeed, we demonstrated that VN/14-1 inhibited MCF-7 tumour growth in a dose-dependent fashion. A dose of 66.0 *μ*mol kg^−1^ day^−1^ caused a reduction of 92.6% in the mean final tumour weight compared with that of tumour-bearing mice receiving vehicle alone, with no apparent toxicity as there was no change in body weight of the mice. It was recently reported that the growth of murine oestrogen-independent TA3-Ha mammary tumours were significantly inhibited by a non-retinoidal RAMBA, R116010 (Van Huesden *et al*, 2002). The study demonstrated the antitumour efficacy of R116010 in an oestrogen-independent model of un-established tumours and it is not clear if the agent is effective against oestrogen-dependent breast tumours. Our findings that VN/14-1 is efficacious against well-established breast tumours suggest that the molecule may be a more effective anticancer RAMBA.

In the present study, we also demonstrated for the first time a synergistic inhibitory effect of VN/14-1 and ATRA on the growth of established MCF-7 and MCF-7Ca breast cancer tumour xenografts *in vivo*, and also the prevention of MCF-7Ca tumour formation. Importantly, VN/14-1 was as effective as the clinically used AIs, anastrozole and letrozole. Both treatments with VN/14-1 caused significant reduction in uteri weights. This suggests that VN/14-1, unlike tamoxifen, may not cause endometrial hyperplasia in patients.

The growth inhibitory effects of cell lines and tumour xenografts especially by VN/14-1 appear to be due to its ability to induce differentiation, apoptosis and cycle arrest. However, in the antitumour efficacy studies VN/14-1 did not cause any tumour regressions. The mechanism underlying the lack of tumour regression is unknown at this time. One possibility is that the treatment with VN/14-1 results mainly in cytostatic but not cytotoxic antitumour effect.

In conclusion, our results clearly suggest that our RAMBAs induce other biochemical pathways in addition to differentiation. It appears that the molecular mechanisms for the antiproliferative activities of our RAMBAs in these breast cancer cells include their ability to induce cell differentiation, apoptosis and effects on cell cycle. Among the RAMBAs tested, VN/14-1 was shown to be the most efficacious in both *in vitro* and *in vivo* studies. Not only are RAMBAs effective against established tumours, but may also be important as differentiating agents in preventing breast cancer. As most cancers become resistant to agents that act on specific targets, the development of molecules that act on multiple cellular targets offer considerable hope for the development of new cancer therapies. These novel RAMBAs endowed with multiple biological activities have potential as therapeutics for breast cancer and possibly other diseases responding to retinoids. Indeed, VN/14-1 has been selected for further preclinical studies. We were recently awarded an Australian patent (Njar *et al*, 2005) to protect these novel and promising anticancer agents and several other patents are pending.

## Figures and Tables

**Table 1:** Inhibitory concentrations (IC_50_ values) of RAMBAs and reference compounds, ATRA and 4-HPR, on the growth of human breast cancer cells

	**Cellular IC_50_ (nM)^a^^,^^b^**		
**Compound**	**MCF-7**	**T47D**	**MDA-MB-231**
VN/14-1	493.5	3.0	24 000.0
VN/50-1	125.9	9.0	—
VN/66-1	590.5	569.9	5620.0
VN/69-1	609.0	—	—
			
*For comparison*			
ATRA	585.2	8.0	22 000.0
4-HPR	147.8	—	1402.0

ATRA=all-*trans*-retinoic acid; RAMBAs=retinoic acid metabolism blocking agents; 4-HPR=4-hydroxyphenyl retinamide.

a Data from Patel *et al*. (2004).

b The IC_50_ values were determined from dose–response curves (by a nonlinear regression analysis using GraphPad Prism) compiled from at least two independent experiments and represents the compound concentration (nM) required to inhibit cell proliferation by 50%; — = not determined.

**Table 2:** Binding activities (IC_50_ values) and transactivation activities (EC_50_ values) of RAMBAs to CRABPs, RARs and RXR*α*

**RAMBAs**	**CRAPBI**	**CRABPII**	**RAR*α***		**RAR*β***		**RAR*γ***		**RXR*α***
	IC_50_^a^	IC_50_^a^	IC_50_^a^	EC_50_^a^	IC_50_^a^	EC_50_	IC_50_^a^	EC_50_	IC_50_^a^
VN/14-1	NB^b^	NB^b^	16	300	200	1500	16	1000	NB^b^
VN/50-1	NB^b^	NB^b^	NB^c^	>10 000	NB^c^	10 000	NB^c^	>10 000	NB^b^
VN/66-1	NB^b^	NB^b^	NB^c^	>10 000	NB^c^	10 000	NB^c^	>10 000	NB^b^
VN/69-1	NB^b^	NB^b^	NB^c^	>10 000	NB^c^	>10 000	NB^c^	>10 000	NB^b^
									
*For comparison*									
	*K*_d_ a	*K*_d_ a	*K*_d_ a	EC_50_^a^	*K*_d_ a	EC_50_^a^	*K*_d_ a	EC_50_^a^	*K*_d_ a
ATRA	4	60	2	20	2	20	2	20	ND
9-CRA	ND	ND	ND	ND	ND	ND	ND	ND	7

CRABPs=cellular retinoic acid binding proteins; RAMBAs=retinoic acid metabolism blocking agents; RARs=retinoic acid receptors; RXR*α*=retinoid X receptors-*α*; 4-HPR=4-hydroxyphenyl retinamide.

a Values are indicated as nM.

b NB=No binding up to 500 nM.

c NB=No binding up to 1000 nM. ND=not determined.

IC_50_ values are the concentration of each VN compound that reduced binding of either [^3^H]-all-*trans*-RA (CRABPs and RARs) or [^3^H]-9-*cis*-RA (RXR*α*) by 50%. These values were determined from dose–response curves compiled from at least three independent experiments.The EC_50_ value for each RAMBA represents the concentration of the compound that results in 50% of the maximal activity obtained with 10^−^6 M all-*trans*-RA. These were also determined from dose–response curves compiled from at least two independent experiments. Details of these experiments are described under Materials and Methods.

**Table 3:** Effects of RAMBAs, ATRA and 4-HPR on T47D cell cycle distribution

**Treatment**	**Sub-G1 (%)**	**Go/G1 (%)**	**S (%)**	**G2/M (%)**
Control	8.89±1.59	74.28±3.15	20.42±1.02	5.31±2.13
VN/14-1	22.38±2.95	77.98±2.56	1.18±1.0	20.85±3.75
VN/50-1	21.67±1.56	77.72±4.54	0.51	21.78±5.05
VN/66-1	26.41±1.18	80.44±0.20	0	19.57±0.21
VN/69-1	24.34±1.24	78.99±0.99	0	21.01±0.99
ATRA	17.12±4.06	83.89±0.96	3.1±1.3	13.03±4.06
4-HPR	30.25±6.66	76.94±3.47	6.32±0.93	16.75±4.39

ATRA=all-*trans*-retinoic acid; RAMBAs=retinoic acid metabolism blocking agents; 4-HPR=4-hydroxyphenyl retinamide. T47D cells were treated with 5 *μ*M of ATRA or RAMBAs for 6 days, or 4-HPR for 4 days. Percentage distribution of cells in each of the cell cycle phases are expressed as mean±s.e. of at least two independent experiments.
